# Comparison of loss of correction between PSO and VCD technique in treating thoracolumbar kyphosis secondary to ankylosing spondylitis, a minimum 2 years follow-up

**DOI:** 10.1186/s13018-019-1170-5

**Published:** 2019-05-16

**Authors:** Yao Wang, Chao Xue, Kai Song, Tianhao Wang, Wenhao Hu, Fanqi Hu, Yongyu Hao, Zhifa Zhang, Chunguo Wang, Xiaoxi Yang, Tianqi Fan, Guoquan Zheng, Zheng Wang, Yan Wang, Xuesong Zhang

**Affiliations:** 1 Chinese PLA General Hospital, 28th Fuxing Road, Beijing, China; 2 Peking University 3rd Hospital, No 49. North Garden Street, Beijing, China

**Keywords:** Correction loss, Pedicle subtraction osteotomy, Vertebral column decancellation, Ankylosing spondylitis, Thoracolumbar kyphosis, Complications

## Abstract

**Background:**

Pedicle subtraction osteotomy (PSO) and vertebral column decancellation (VCD) are frequently used methods for correction of thoracolumbar kyphosis resulting from ankylosing spondylitis (AS). However, there are limited reports performed to evaluate the difference of loss of correction and the effectiveness of PSO and VCD techniques in patients with thoracolumbar kyphosis secondary to AS.

**Objective:**

To retrospectively estimate the effectiveness of correction and loss of correction of PSO and VCD techniques in patients with thoracolumbar kyphosis secondary to AS.

**Methods:**

We performed a retrospective review of 61 consecutive AS kyphosis patients undergoing PSO or VCD surgery from March 2012 to April 2015. The patients were divided into PSO group (*n* = 25) and VCD group (*n* = 36) according to the types of osteotomies. Measurement of the radiographic parameters was performed and the change was analyzed.

**Results:**

Mean loss of correction in the global kyphosis was 2.31° in the PSO group and 2.29° in VCD group at the last follow-up, respectively, with no significant difference. Progressive junctional kyphosis occurred in both groups. VCD obtained a significantly larger correction than PSO while sharing a similar incidence of complications. No serious complications were observed in the two groups.

**Conclusion:**

The PSO osteotomy and VCD osteotomy are both safe and effective methods in treating thoracolumbar kyphosis secondary to AS. Mild loss of correction mainly occurred in the global kyphosis in both techniques with no significant difference.

## Introduction

Ankylosing spondylitis (AS) is a chronic inflammatory disease that primarily affects the spine and sacroiliac joints [1]. A significant proportion of patients afflicted with ankylosing spondylitis may suffer from debilitating, progressive stiff kyphosis of the thoracolumbar spine [1, 2]. Despite improving medical therapies and conservative treatment modalities, there still exist specific clinical indications for surgical correction of thoracolumbar kyphotic deformities in this patient population to restore the sagittal balance and the ability to see straight ahead and to improve diaphragmatic respiration and decrease pressure on the abdominal cavity. Currently, various osteotomy techniques have been applied to treat AS spinal deformities including pedicle subtraction osteotomy (PSO) and vertebral column decancellation (VCD), which are frequently used for correction of thoracolumbar kyphosis resulting from AS [3]. Some previous studies have analyzed the indication, technical aspects, correction obtained, and complication rates of the aforementioned techniques [4–6]. To our best knowledge, there are limited reports addressing a comparison of loss of correction between PSO and VCD technique in kyphotic deformity resulting from AS. Therefore, this retrospective study was conducted to estimate the effectiveness of correction and loss of correction of PSO and VCD techniques in patients with thoracolumbar kyphosis secondary to AS.

## Materials and methods

### Design

We conducted a retrospective study of patients with a diagnosis of ankylosing spondylitis with thoracolumbar kyphosis who underwent corrective surgery in our center between March 2012 and April 2015. The inclusion criteria involved (1) preoperative global kyphosis (GK) ranging from 40 to 120°, (2) no scoliosis or with a coronal curve < 20°, (3) underwent a single level PSO or VCD surgery, (4) with a minimum 2-year follow-up [7]. The patients who had previous spinal surgeries or complicated with Andersson lesion were excluded in this study. Informed consent was obtained from all participants, and procedures were conducted according to the Declaration of Helsinki.

### Patients

In the present study, a total of 61 AS patients consisting of 57 males and 4 females were included eventually (Table 1). On the basis of the types of osteotomies, 61 patients were divided into two groups: (1) PSO group (*n* = 25) included 23 male and 2 female patients, with an average age of 38.5 ± 11.5 (range 22–63 years) years. Mean follow-up was 30.2 ± 4.47 (range 24–38 months) months. The preoperative GK was 62.26 ± 14.80° (range 42.84–92.61°); (2) VCD group (*n* = 36) consisted of 34 male and 2 female patients, with average age of 38.1 ± 9.0 (range 21-54 years) years. Mean follow-up was 28.8 ± 3.7 (range 24–39 months) months. The preoperative GK was 76.43 ± 17.45 (range 48.66–118.24°) degrees.

**Table 1:** Patients’ demographic data (mean ± SD, range)

Parameters	PSO group (*n* = 25)	VCD group (*n* = 36)
Age (years)	38.5 ± 11.5 (22–63)	38.1 ± 9.0 (21–54)
Gender	23 males, 2 females	34 males, 2 females
Follow-up (months)	30.2 ± 4.7 (24–38)	28.8 ± 3.7 (24–39)

### Surgical techniques

All patients were continuously monitored intraoperatively by somatosensory-evoked potentials and motor-evoked potentials. Following the induction of general anesthesia, each patient was placed in the prone position. The thoracolumbar spine was exposed through a posterior midline incision, and the posterior elements were exposed through a subperiosteal approach. Pedicle screw fixation was performed by a freehand technique.

**Fig. 1:**
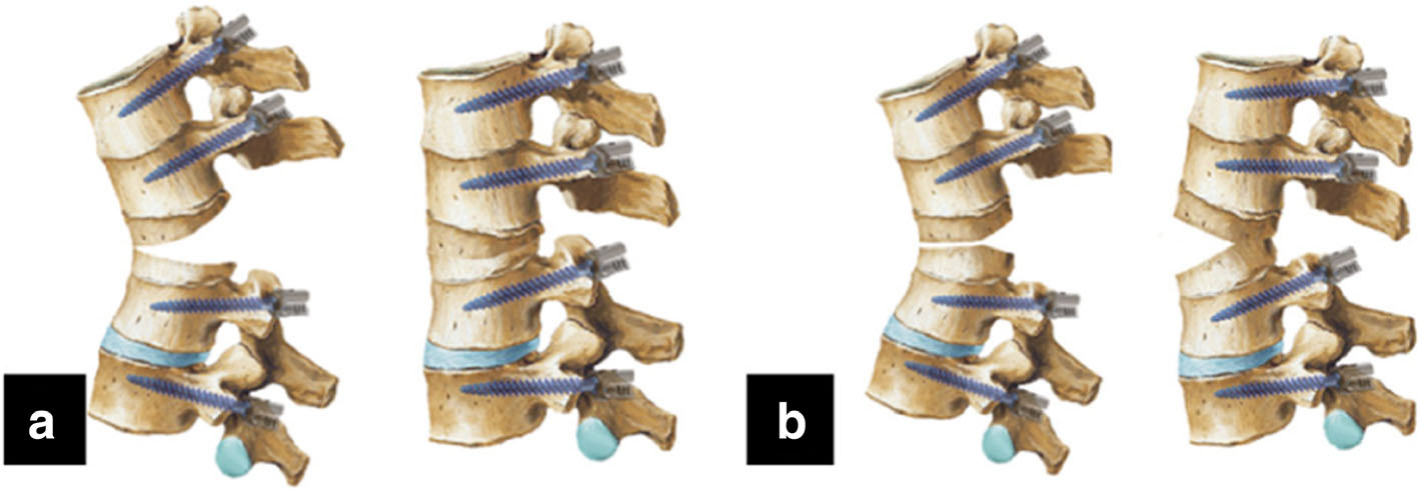
Spine osteotomy techniques. **a** Pedicle subtraction osteotomy. **b** “Y”-shaped vertebral column decancellation.

Both osteotomies were initiated by probing the pedicles of the osteotomized vertebrae on both sides using a pedicle probe. In PSO osteotomy, the pedicles were dilated by incrementally increasing the probe or bone tap size. A high-speed drill was used to enlarge the pedicle holes as necessary. After this, the vertebral bone was adequately removed. The decancellation procedure carefully created a triangular wedge to the anterior cortex. Care must be taken to protect the nerve root and the contiguous vessels. In VCD osteotomy, the procedure is a “Y”-type osteotomy rather than a “V”-type osteotomy in PSO technique (Fig. 1). The VCD technique was characterized by controlled anterior column opening, posterior column closing, and middle column preservation as the hinge. The key points of the “Y”-shaped VCD technique are to remove a relatively small amount of the posterior half of the osteotomy column and preserve as much as possible of the middle column as the hinge, which serves as the correction “leverage” to provide greater stability and to prevent sagittal translation during the correction procedure.

Finally, the closure of osteotomy space was performed by gentle pressure on the pedicle screws above and below the osteotomy with precontoured rods used to strengthen the stability of the osteotomy site. The patient’s position and the operating table were simultaneously adjusted. After closure, evoked potentials were performed to assess spinal cord and nerve root function. Then, a posterior fusion bed was prepared, and the local bone was grafted to create a fusion. C-arm fluoroscopy was used to examine the magnitude of the correction. Postoperatively, the patients were allowed to mobilize using a well-molded thoracolumbosacral orthosis during the first 3 months.

### Radiographic measurements

**Fig. 2:**
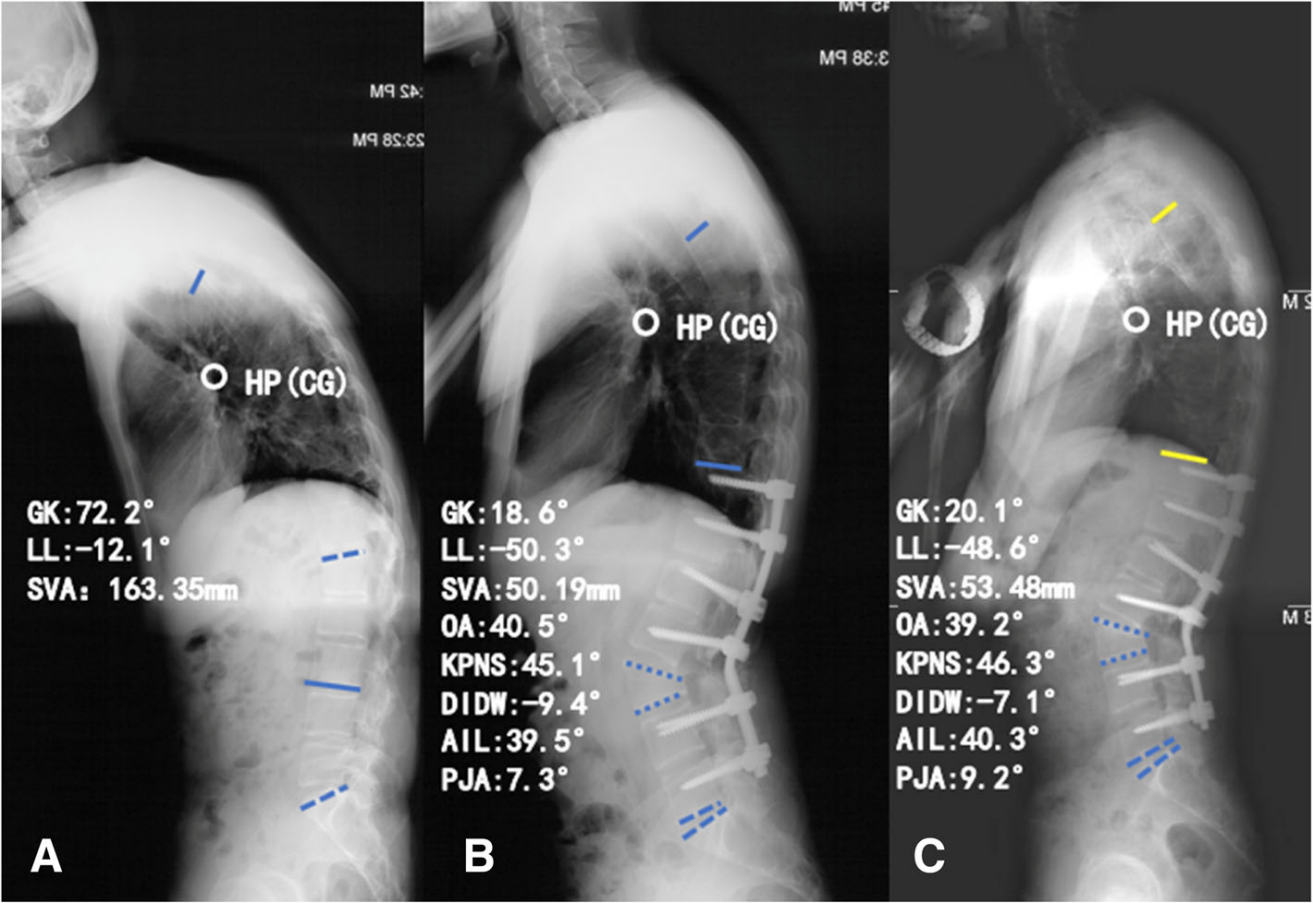
A 37-year-old male patient with thoracolumbar kyphosis secondary to ankylosing spondylitis. **a** Preoperative standing lateral radiograph. **b** Immediately after surgery with pedicle subtraction osteotomy (PSO) technique. **c** Lateral radiograph taken 26 months after surgery. AIL angle of instrumented levels, DIDW the sum of distal non-fused intervertebral disc wedging, GK global kyphosis, KPNS kyphotic angle of proximal non-fused segment involved in the global kyphosis, LL lumbar lordosis, OA osteotomized vertebra angle, PJA proximal junctional angle, SVA sagittal vertical axis, HP hilus pulmonis, CG the center of gravity

**Fig. 3:**
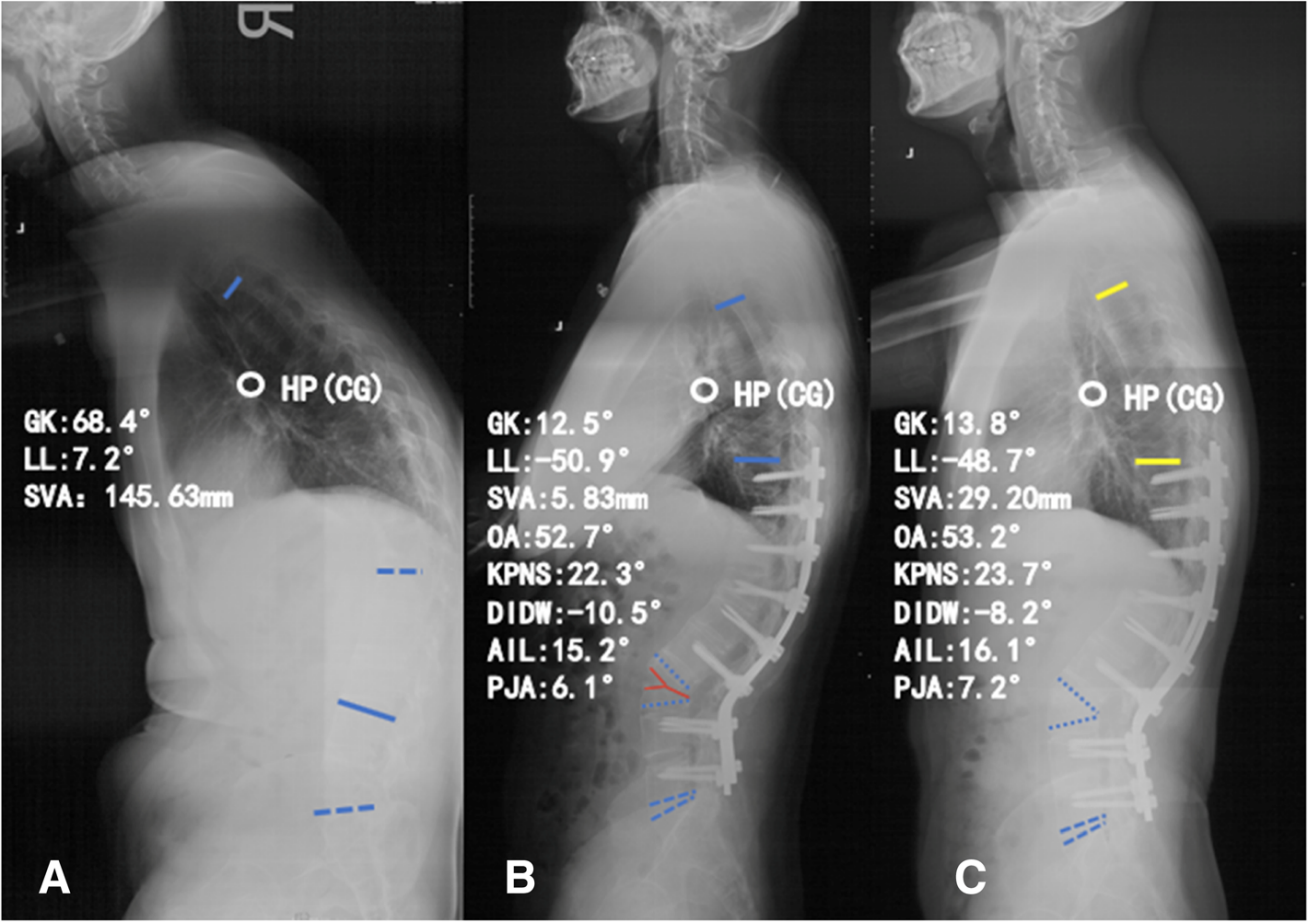
A 38-year-old male patient with thoracolumbar kyphosis secondary to ankylosing spondylitis. **a** Preoperative standing lateral radiograph. **b** Immediately after surgery with vertebral column decancellation (VCD) technique. **c** Lateral radiograph taken 32 months after surgery. AIL angle of instrumented levels, DIDW the sum of distal non-fused intervertebral disc wedging, GK global kyphosis, KPNS kyphotic angle of proximal non-fused segment involved in the global kyphosis, LL lumbar lordosis, OA osteotomized vertebra angle, PJA proximal junctional angle, SVA sagittal vertical axis, HP hilus pulmonis, CG the center of gravity

**Fig. 4:**
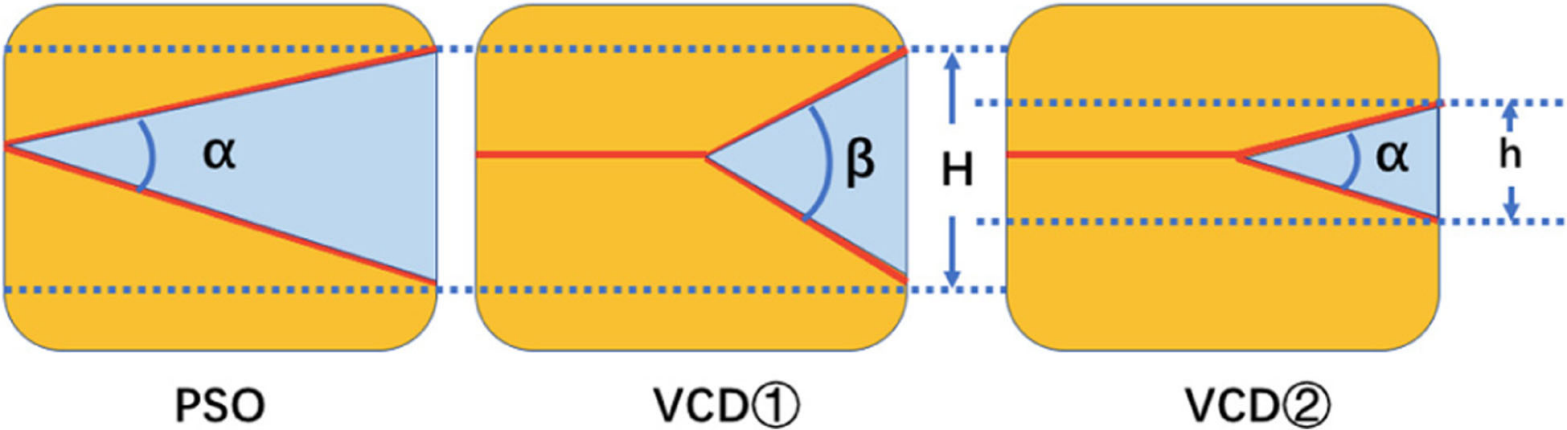
Illustration of the differences of PSO and VCD technique. By shortening the same height of posterior vertebral column (H), the VCD osteotomy may achieve larger correction (*β*) comparing with PSO technique (*α*). To obtain a same corrective angle (*α*), the shortening of posterior vertebral column in VCD osteotomy (h) is much smaller than that in PSO technique (H)

Anteroposterior and lateral full-length X-ray images from patients in a freestanding posture were obtained before and after corrective surgery, before discharge from the hospital (defined as early postoperative) and minimum 2 years postoperatively. On these images, we measured spinal parameters including (1) sagittal vertical axis (SVA) [8], the distance measured between the C7 plumb line and the posterosuperior corner of S1 vertebra; (2) global kyphosis (GK) [9], the angle between the superior endplate of the maximally tiled upper end vertebra and the inferior endplate of the maximally tilted lower end vertebra (Fig. 2a); (3) lumbar lordosis (LL) [10], the angle between the superior endplate of L1 and S1, positive value indicates lumbar kyphosis and negative value indicates lumbar lordosis (Fig. 2a); (4) angle of instrumented levels (AIL), the Cobb angle between the upper end plate of the proximal fixed segment and the lower end plate of the distal-fixed segment. In the same way, negative value indicated lordosis, whereas positive value demonstrated kyphosis (Fig. 2b); (5) osteotomy angle (OA), the changed angle between superior and inferior endplate of osteotomized vertebra (Fig. 2b); (6) proximal junctional angle (PJA) [11], determined by the angle between the caudal endplate of the upper instrumented vertebra and the cranial end plate of two vertebrae above. Proximal junctional kyphosis (PJK) was defined as a kyphosis of the junctional area which was > 10° and at least 10° greater than the preoperative measurement [12, 13]; (7) kyphotic angle of proximal non-fused segment involved in the GK (KPNS) [14]: the angle between the superior endplate of the preoperative maximally tilted upper end vertebra and the superior endplate of upper end vertebra of the fused segments (Fig. 2b); and (9) the sum of distal non-fused intervertebral disc wedging (DIDW) (Fig. 2b). The angle is positive if the curve is kyphotic and negative if the curve is lordotic in LL [14]. All of the parameters were measured by an orthopedic resident three times using surgimap software (Nemaris, New York, NY) and the average values were calculated (Fig. 3).

### Statistical analysis

The analyses were performed with the Statistical Package for the Social Sciences version 22.0 (SPSS Inc., Chicago, IL, USA). The paired sample *t* test was run for intragroup comparison, whereas an independent sample *t* test was used to compare differences between the two groups. *p* < 0.05 was considered significant.

## Results

### Operative procedure

All patients (*n* = 61) in both groups received one-level osteotomy. In the PSO group (*n* = 25), the level of osteotomy was located at L1 in 4 cases, L2 in 14 cases, and L3 in 7 cases. The instrumented fusion involved 5 to 12 segments, with an average of 6.6 segments. As for the VCD group, the osteotomy was performed at L1 in 5 cases, L2 in 19 cases, and L3 in 12 cases. The instrumented fusion segments involved 5 to 10 segments, with an average of 6.5 segments. The mean intraoperative blood loss was 970 mL (range 700–1500 mL) in the PSO group and 872 mL (range 550–1400 mL) in the VCD group, respectively, showing the same blood loss in VCD patients compared with PSO patients (*p* = 0.102).

### Complications

Dura tears were observed during lamina resection in two cases in the PSO group, while three cases in the VCD group. All of them were recovered without adverse effects at the final follow-up. The intraoperative somatosensory-evoked potentials monitoring showed a decrease in one case in both groups, which had transient lower extremities weakness and recovered completely at the follow-up of 6 months. All the patients felt abdominal muscles tense, and seven (two in the PSO group and five in the VCD group) suffered from tensive lesions, which were all recovered 2 weeks later. No paralytic ileus occurred in this study. Mild sagittal translation occurred in seven patients (three in the PSO group and four in the VCD group) at the osteotomy sites with no neurological deficit. PJK was observed in both groups (one in the PSO group and one in the VCD group). No fixation failure or main vascular injury was detected at the follow-up (Table 2).

**Table 2:** Complications in two groups

Complications	PSO group (*n* = 25)	VCD group (*n* = 36)
Dural tears	2 (8%)	3 (8.3%)
Transient lower extremity weakness	1 (4%)	1 (2.8%)
Abdominal tensive lesions	2 (8%)	5 (13.9%)
Ileus	0	0
Mild sagittal translation	3 (12%)	4 (11.1%)
PJK	1 (4%)	1 (2.8%)
Fixation failure	0	0
Main vascular injury	0	0

### Radiological results

Postoperative correction was achieved in all the patients immediately. Solid fusions were achieved in all the patients according to radiological evidence, and no pseudarthrosis was observed at the osteotomy level in either group at the last follow-up visit. The radiographic measurements are shown in Table 3.

**Table 3:** Radiographic assessment of preoperative, postoperative and the final follow-up data

Parameters	Group	Preoperative	Postoperative	*p*	Final follow-up	*p*	Correction	Loss of correction
GK (°)	PSO	62.26 ± 14.80*	24.61 ± 12.99^a^	*< 0.001*	26.92 ± 16.00^b^	*< 0.05*	37.65 ± 9.16*	2.31 ± 6.23
	VCD	76.43 ± 17.45*	27.31 ± 14.01^a^	*< 0.001*	29.60 ± 14.64^b^	*< 0.05*	49.13 ± 9.87*	2.29 ± 4.02
LL (°)	PSO	-1.48 ± 8.32	-35.18 ± 25.84*^a^	*< 0.001*	-34.47 ± 24.69*	*0.338*	33.69 ± 24.08*	-
	VCD	1.15 ± 8.74	-43.78 ± 13.62*^a^	*< 0.001*	-43.74 ± 13.36*	*0.914*	44.93 ± 11.73*	-
SVA (mm)	PSO	170.94 ± 36.98	58.37 ± 25.77^a^	*< 0.001*	62.42 ± 25.72	*0.265*	112.57 ± 29.36	-
	VCD	173.54 ± 55.30	62.71 ± 48.15^a^	*< 0.001*	64.44 ± 49.39	*0.574*	110.83 ± 35.68	–
OA (°)	PSO	–	33.84 ± 5.23*	–	33.70 ± 5.51*	*0.659*	–	–
	VCD	–	44.10 ± 8.14*	–	43.95 ± 7.97*	*0.430*	–	–
KPNS (°)	PSO	–	30.58 ± 11.62	–	34.49 ± 12.06^b^	*< 0.001*	–	–
	VCD	–	32.19 ± 11.98	–	35.60 ± 13.49^b^	*< 0.001*	–	–
DIDW (°)	PSO	–	-6.45 ± 4.75	–	-6.32 ± 4.95	*0.872*	–	–
	VCD	–	-8.26 ± 4.22	–	-7.98 ± 3.67	*0.640*	–	–
AIL (°)	PSO	–	19.76 ± 14.47	–	19.56 ± 13.85	*0.454*	–	–
	VCD	–	13.11 ± 8.42	–	13.42 ± 7.81	*0.200*	–	–
PJA (°)	PSO	–	11.88 ± 5.51	–	14.24 ± 6.86^b^	*< 0.001*	–	–
	VCD	–	14.03 ± 6.96	–	16.33 ± 7.27^b^	*< 0.001*	–	–

*AIL* angle of instrumented levels, *DIDW* the sum of distal non-fused intervertebral disc wedging, *GK* global kyphosis, *KPNS* kyphotic angle of proximal non-fused segment involved in the global kyphosis, *LL* lumbar lordosis, *OA* osteotomized vertebra angle, *PJA* proximal junctional angle, *PSO* pedicle subtraction osteotomy, *SVA* sagittal vertical axis, *VCD* vertebral column decancellation

*Significant difference between 2 groups: *p* < 0.05

^a^Significant difference between preoperative and postoperative values: *p* < 0.05

^b^Significant difference between postoperative and final follow-up values: *p* < 0.05

In both groups, the sagittal balance was significantly improved immediately after surgery including the GK (*p* < 0.001), LL (*p* < 0.001), and SVA (*p* < 0.001). The magnitude of correction in VCD group was significantly larger than that in the PSO group (*p* < 0.001). At the final follow-up, SVA and LL remained in both groups (*p* > 0.05). However, between the immediate postoperative and final follow-up assessments, the mean GK increased from 24.61 to 26.92° (*p* = 0.026) with an average loss of correction of 2.31° in the PSO group and from 27.31 to 29.60° (*p* = 0.002) with a mean loss of correction of 2.29° in the VCD group. The correction of the osteotomy sites, which the OA reflects, maintained till the final follow-up with about 34° in the PSO group (*p* = 0.659) and approximately 44° in the VCD group (*p* = 0.430) and were significantly different between the two groups (*p* < 0.001). KPNS and PJA were slightly increased while DIDW and AIL remained at the last follow-up in both groups.

## Discussion

Traditional PSO technique is a three-column closing-wedge osteotomy. During this aggressive procedure, posterior elements and a V-shaped bony wedge of the vertebral body are resected. Many researchers believe excessive shortening is dangerous and that a safety limitation exists [15, 16]. Gertzbein and Harris [17] limited their corrections to approximately 30 to 40°. If the kyphotic correction is larger than 40° with PSO, the spinal cord may be too long for the shortened column, and the cord may become curved or kinked or potentially damaged because the hinge is positioned at the anterior longitudinal ligament at the apex of the deformity [18]. In order to overcome the limitations of previous techniques including PSO, while merging benefits of each technique, “Y”-shaped VCD osteotomy was developed and introduced by Wang [19], which was characterized by controlled anterior column opening, posterior column closing, and middle column preservation as the hinge. Theoretically, compared with the PSO technique, the VCD osteotomy may achieve larger correction by shortening the same height of the posterior vertebral column. In other words, to obtain the same corrective angle, the shortening of the posterior vertebral column in VCD osteotomy is much smaller than that in the PSO technique (Fig. 4). In the present study, the OA of VCD group with an average of 44.10°, which indicates the correction of the osteotomy site, is approximately 10° larger than that of the PSO group with a mean of 33.84° (Table 3).

Several previous studies have focused on the loss of correction of kyphotic deformity secondary to AS. Chang [18] performed a research to compare lumbar SPO and PSO on single-level in patients with thoracolumbar kyphotic deformity result from AS and found similar correction loss in both groups. In Zhu’s study [7], loss of correction in the GK of SPOs technique (3.9°) and PSO technique (2.4°) was identified 2 years after surgery. More recently, Mu [14] conducted a retrospective research in PSO-treated AS patients using comprehensive methods in analyzing the radiographic parameters and found a worsening of GK (2.82°) combined with decreased LL (3.77°) at the last visit of 6.9 years postoperatively. To our best knowledge, it remains unclear of the difference between PSO and VCD techniques on correction loss in long-term follow-up.

In this study, the two techniques were used in AS-related thoracolumbar kyphosis, and we retrospectively compared the efficacy of two groups with a minimum of 2 years follow-up. Higher improvement of sagittal balance, including GK and LL, was achieved comparing the preoperative and immediate postoperative period in VCD group than that in the PSO group. On average, a correction about 38° in GK and 34° in LL was achieved in the PSO group, while approximately 50° in GK and 45° in LL in the VCD group. The SVA decreased from approximately 171–174 mm before the surgery to average 59 mm in the PSO group and 63 mm in the VCD group after surgery and maintained till the final follow-up. Despite the immediate satisfactory surgical results, mild loss of correction of GK was observed in both groups in the long-term follow-up with no significant difference, which was in line with the previous studies [7, 14]. However, different from Qiao’s study [14], no significant changes were observed in the correction loss of lumbar lordosis. In the range of the lumbar lordosis, the angle of DIDW might randomly change based on the remained flexibility of the caudal unfused segments, while the instrumented segments (AIL) remained stable. Similar to McMaster and Coventry’s conclusion [20] that was obtained from a 10-year follow-up in 17 patients, once the osteotomy had fused, correction in the lumbar region was maintained.

According to Zhu’s theory [7], in order to maintain the correction and ensure rapid fusion, the weight-bearing line should be placed through or posterior to the sacrum. In 2013, Song et al. [21] considered the hilus pulmonis to be a marker as the center of gravity of the trunk for AS thoracolumbar kyphosis. In our observation, when the hilus pulmonis as the center of gravity was located above the osteotomy area, the correction could remain well. Among the three cases with correction loss > 5°, the hilus pulmonis as the center of gravity were all located in the front of the osteotomy site.

In fact, although the osteotomy surgery obtained a better sagittal balance for the patients in an upright position, the patients often have a tendency for forward leaning in daily life, such as eating, picking up things, and tying shoelaces. Under these circumstances, the stress at the junctional area between the instrumented and non-instrumented segments might increase, favoring the formation of progressive junctional kyphosis and correction loss, which was reflected by the PJA in the present study. PJK developed in one patient in both of the groups. The one in the PSO group had revision surgery with extension of the fusion and instrumentation to the upper thoracic spine because of back pain occurrence. Based on our experience to prevent PJK, there are four main points: (1) the cephalad end of the prebent rod should be shaped adjusting to the curvature of the proximal spine, (2) the upper instrumented vertebra (UIV) should not be selected at the kyphosis apex or stress concentration area, especially when the patient is suspected of Andersson lesion, (3) anti-inflammation medication should be regularly applied to prevent progression of active disease and loss of correction, and (4) avoid heavy weight lifting after surgery.

Compared with the PSO, the VCD technique has a similar incidence of perioperative and long-term complications (Table 2). Dura laceration mainly occurred in the lamina resection because of the dural adhesions to the ossified ligamentum flavum and was repaired with gel sponge. Transient lower extremity weakness appeared in two patients, which were limited to one side and predicted with spinal cord monitoring. When SEP or MEP prompted a neurologic deficit, the procedure of closing the osteotomy space was stopped, and the examination of the nerve root and decompression was performed. All of them recovered completely at the follow-up of 6 months. Mild sagittal translation occurred during the closing of the osteotomy space. Posterior element decompression was performed when translation was found. Presently, we observed that abdominal tensive lesions increased with VCD, perhaps in association with the larger correction in these patients. The lesions subsided several days after covering the cutaneous area with a magnesium sulfate sponge, and all recovered in 2 weeks. No life-threatening complications were observed in the two groups.

Although the loss of correction in the PSO and VCD groups was compared in detail and the effectiveness of correction of both techniques was estimated, the limitations of this research should be mentioned. One of them is that the present study was designed retrospectively and the sample size was relatively small. While the spinopelvic balance is quite important, assessment of the pelvic parameters has not been addressed. Further study with a longer follow-up of this issue is warranted in the future.

## Conclusions

In summary, the PSO osteotomy and VCD osteotomy are both safe and effective methods in treating thoracolumbar kyphosis secondary to ankylosing spondylitis. Mild loss of correction mainly occurred in the global kyphosis and showed no significant difference in both groups. Locating the hilus pulmonis as the center of gravity above the osteotomy area may help to remain the correction. While sharing a similar incidence of complications, an average of 44.10° correction was obtained by VCD osteotomy which was approximately 10° larger than that of the PSO group with a mean of 33.84°.

## Abbreviations

AIL: Angle of instrumented levels
AS: Ankylosing spondylitis
DIDW: The sum of distal non-fused intervertebral disc wedging
GK: Global kyphosis
KPNS: Kyphotic angle of proximal non-fused segment involved in the GK
LL: Lumbar lordosis
OA: Osteotomy angle
PJA: Proximal junctional angle
PJK: Proximal junctional kyphosis
PSO: Pedicle subtraction osteotomy
SVA: Sagittal vertical axis
UIV: The upper instrumented vertebra
VCD: Vertebral column decancellation
